# Supplementary material for: A Critical View of the Application of the APEX Software (Aqueous Photochemistry of Environmentally-Occurring Xenobiotics) to Predict Photoreaction Kinetics in Surface Freshwaters
Source: Molecules. 2019 Dec 18;25(1):9. doi: 10.3390/molecules25010009 (PMC7017383; doi:10.3390/molecules25010009)
Supplement: Supplementary file 1 [file molecules-25-00009-s001.zip › 3.2.4_gcc-4.4.0.zip!/3.2.4_gcc-4.4.0/3.2.4_gcc-4.4.0/doc/octave/html/interpreter/The-_003ccode_003etry_003c_002fcode_003e-Statement.html]

The @code{try} Statement - Untitled


Next: Continuation Lines,
Previous: The <code>unwind\_protect</code> Statement,
Up: Statements


---

### 10.9 The `try` Statement

In addition to unwind\_protect, Octave supports another limited form of
exception handling.

The general form of a `try` block looks like this:

```
     try
       body
     catch
       cleanup
     end_try_catch
```

where body and cleanup are both optional and may contain any
Octave expressions or commands. The statements in cleanup are
only executed if an error occurs in body.

No warnings or error messages are printed while body is
executing. If an error does occur during the execution of body,
cleanup can use the function `lasterr` to access the text
of the message that would have been printed. This is the same
as `eval (`try`,` catch`)` but it is more efficient since
the commands do not need to be parsed each time the try and
catch statements are evaluated. See Errors and Warnings, for more
information about the `lasterr` function.
